# Amniotic fluid characteristics and its application in stem cell therapy: A review

**DOI:** 10.18502/ijrm.v20i8.11752

**Published:** 2022-09-06

**Authors:** Hoda Shamsnajafabadi, Zahra-Soheila Soheili

**Affiliations:** National Institute of Genetic Engineering and Biotechnology, Tehran, Iran.

**Keywords:** Amniotic fluid, Stem cells, Differentiation, Regeneration, Tissue engineering.

## Abstract

Amniotic fluid (AF) is a clear yellow fluid that surrounds the fetus during pregnancy. The amniotic sac consists of 2 layers: the amnion and the chorion. Osmotic and hydrostatic forces cause the maternal plasma to pass through the fetal skin and generate the AF. AF allows the fetus to grow inside the uterus, supports it from injuries, retains consistent pressure and temperature, and enables the exchange of body chemicals with the mother. At first, it consists of water and electrolytes but after the 12-14 ${}^{\text{th}}$ wk the liquid also contains carbohydrates, proteins, lipids, phospholipids, urea, hormones, and some biochemical products. AF appearance is characterized by the grade of cloudiness and the number of flakes of the vernix. The volume of AF increases with the fetus's growth. Its appearance depends on the gestational age. In addition to differentiated cells, stem cells are also found within the AF. These cells express embryonic-specific cell markers and bear high self-renewal capacity and telomerase activity. AF stem cells possess the potential to differentiate into osteogenic, cardiac, skeletal muscle, lung, neuronal, kidney, bone, cartilage, ovarian and hepatic cells in vitro. They represent a great promise in regenerative medicine for the reconstruction of bio-artificial tissues and organs in vivo*. *The purpose of this paper was to briefly review the development and function of AF and the application of its stem cells in cell therapy.

## 1. Introduction

Amniotic fluid (AF) is a complex physiological and dynamic biological fluid that surrounds the fetus during pregnancy. It provides mechanical protection and the nutrients required for fetal growth and wellbeing. After about 12 days of pregnancy, an amniotic membrane forms and lines the fetal cavity, while contemporary AF begins to fill the amniotic sac (1). In this review, we explain the features of AF stem cells (AFSCs). We focus on their ability to differentiate in vitro and their potential application for tissue regeneration in vivo.

### AF sources

In the first trimester of pregnancy, the osmolality of the AF and maternal plasma are the same, which suggests that the maternal plasma generates the AF (2). Until the 22-25 ${}^{\text{th}}$ wk, the fetal skin is non-keratinized which allows AF to penetrate the skin and the surfaces of the placenta, amnion, and umbilical cord, which are freely penetrable to water and solutes (3). After the 24 ${}^{\text{th}}$ wk, the surfaces of the nose and mouth exchange fluid, but this is not the main origin of AF (4). In the latter half of pregnancy, the main origins of AF are from fetal urine production, pulmonary excretion, fetal swallowing, transmembranous movement among the amnion and chorion, and intramembranous movement between the fetal blood and the placenta (Table I) (5-9).

### AF function

AF has vital functions such as nourishment, and antimicrobial and mechanical protection of the fetus during pregnancy. The most important roles of AF are as follows:

1. Shock absorber: AF protects the fetus from mechanical trauma (10).

2. Body parts development: AF helps the growth of the external body parts such as fingers and toes and the development of the lungs and the digestive systems by circulating freely within the womb and lubricating each part of the body.

**Table 1:** The main origins of AF near the full term of pregnancy


**Author, year (Ref)**	**AF origins**	**Amount (ml/day)**	**Direction**
**Van Otterlo ** ***et al*** **., 1977 (7)**	Fetal urine	800-1000	Fetus to AF
**Abramovich ** ***et al*** **., 1979 (8)**	Fetal swallowing	500-1,000	AF to fetus
	Intramembranous pathway	200-500	AF to fetus
	Fetal lung	340	AF to fetus and fetus to AF
**Brace, 1997 (9)**	Transmembranous pathway	10	AF to fetus
	AF: Amniotic fluid			

3. Antibacterial activity: AF plays a significant role in the baby's natural immune system. It contains different antimicrobial substances including lactoferrin, lysozyme, bactericidal/permeability-increasing protein, a-defensins (HNP1-3), psoriasin (S100A7), calprotectin, cathelicidin (LL-37), and secretory leukocyte protease inhibitor, which contribute to the natural immune system in babies. These antimicrobials protect the baby from various bacteria, protozoa, fungi, and viruses (11).

4. Nutrition delivery: AF is rich in carbohydrates, hormones, lipids, lactate, electrolytes, enzymes, proteins, peptides and pyruvate which support fetal growth (4).

5. Temperature stabilizer: AF stabilizes the temperature to protect the fetus from extreme temperature changes (12).

### AF composition

AF initially consists of water from the mother but gradually, a larger proportion is made up of the baby's urine. It also consists of different kind of nutrients, hormones, and biochemical products (Table II) (13-15).

**Table 2:** AF composition of nutrients, hormones and biochemical products


**Author, year (Ref)**	**AF composition**	**Components**
**Underwood ** ***et al*** **., 2005 (4)**	Nutrients	Proteins, amino acids, lactate, pyruvate, peptides, carbohydrates, lipids, phospholipids, urea, and electrolytes
**Dawood, 1977 (13)**	Hormones	Prolactin, growth hormone, human chorionic somatomammotropin, placental hormone, human chorionic corticotropin, human chorionic gonadotropin, human chorionic thyrotropin, and placental luteinizing hormone-releasing factor
**Oliveira ** ***et al*** **., 2002 (14)** **Bauk ** ***et al*** **., 1996 (15)**	Biochemical products	Creatinine, N-acetyl-ß-D-glucosaminidase, urea, potassium, uric acid, ß2-microglobulin, glucose, sodium, phosphorus, calcium, and albumin
AF: Amniotic fluid		

**Figure 1:**
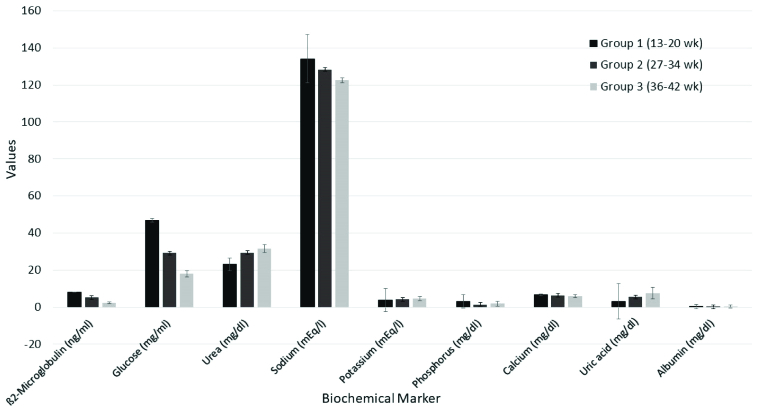
Values of biochemical markers of AF throughout gestation, data was obtained from Oliveira et al., 2002 article (14).

Biochemical products are interchanged between the mother and fetus. The value of some biochemical markers such as ß2-microglobulin, glucose, and uric acid present significant correlations with gestational age and creatinine, while other biochemical markers like urea, potassium, and phosphorus present mild correlations with age and creatinine. ß2-microglobulin, glucose, and uric acid are significant indicators of the function and maturation of fetal kidneys (Figure 1) (14). From the 14 ${}^{\text{th}}$ -16 ${}^{\text{th}}$ wk of gestation, the AF is composed of some growth factors that promote the growth, attachment, polarity, and migration of cells in culture. We previously demonstrated that AF was able to advance the trans differentiation of retinal pigmented epithelium cells into rod photoreceptors, retinal progenitor, and retinal ganglion cells (16-18).

### AF appearance

**Figure 2:**
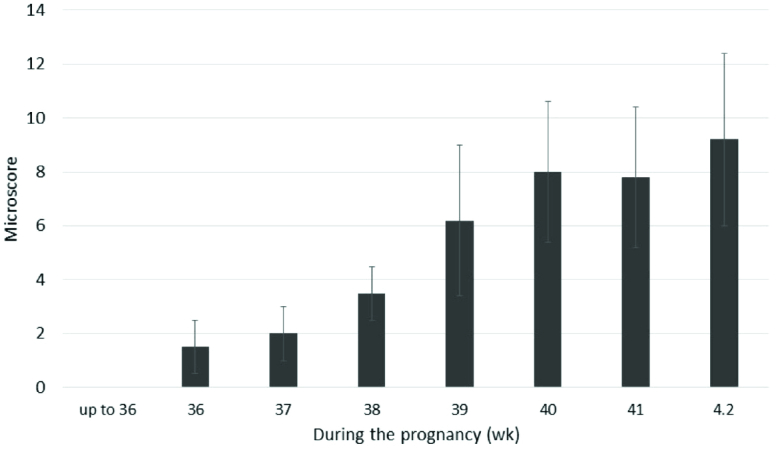
Microscore of AF during pregnancy, data was obtained from Verpoest MJ, 1976 article (19).

AF appearance is determined by the degree of cloudiness and the number of flakes over the pregnancy. Flakes are released from the fetal skin and depend on the maturation of the central nervous system of the fetus. Thus, the appearance of the AF is an indicator of fetal maturity. Macroscore is a system that categorizes the summation of flake quantity and the cloudiness degree as a score ranging from 0-6, from no flakes or no cloudiness to a large number of flakes and very cloudy fluid. Thus, the microscore can fluctuate between 0 and 12 (Figure 2).

At the end of the 32 ${}^{\text{nd}}$ wk of pregnancy, the mean macroscore is 0; the AF is clear and does not have any flakes. By the 36 ${}^{\text{th}}$ wk, the average macroscore is 1-2 as the AF has some flakes and carries a trace of cloudiness. After that the score increases until the 40 ${}^{\text{th}}$ wk of pregnancy; by this wk the mean score is 8 ± 2.6 as the AF contains a moderate amount of flakes and is cloudy. The mean macroscore is about 8 ± 3.2 in the 41 ${}^{\text{st}}$ wk, and thereafter the score increases until 9.3 ± 2.5 in the 42 ${}^{\text{nd}}$ wk. By this wk it has a large number of flakes and is very cloudy (Figure 2) (19).

### AF volume (AFV)

AFV can be measured directly at the time of cesarean or by uterine hysterotomy, or indirectly by dye-dilution techniques or sonography. Because direct and indirect techniques are time-consuming and invasive and may require laboratory support, AFV is commonly estimated by ultrasound (20). The advantages of sonography are that it is easy to carry out and teach, and it can be reproduced (21).

At the 12 ${}^{\text{th}}$ wk of gestation, the average AFV is about 60 ml and by the 16 ${}^{\text{th}}$ wk, it becomes 175 ml. In the 20 ${}^{\text{th}}$ and 30 ${}^{\text{th}}$ wk, there is a volume of 300 and 600 ml, respectively. AFV increases regularly during pregnancy until 400-1200 ml at 34-38 wk. After 38 wk, AFV decreases to 800 ml by the 40 ${}^{\text{th}}$ wk. After 43 wk, this volume diminishes to 250 ml (22).

Oligohydramnios occurs when there is an AFV < 200-500 ml, which is detected by ultrasound (23). This can be due to idiopathic underproduction or loss of fluid. Underproduction can be related to urinary tract obstruction, dysfunctional kidneys, maternal dehydration, or abnormal placental function (5). Loss of AF might happen due to rupture of surrounding membranes (24). Maternal hydration affects idiopathic oligohydramnios (25), so oral hydration is a noninvasive, easily accessible intervention to treat it (26). Oligohydramnios can cause fetal lung hypoplasia and contracture malformations (27). It can also increase the risk of non-reassuring fetal heart rate patterns, cesarean delivery, meconium aspiration, neonatal intensive care unit admission, and mortality (28). It has been estimated that 13.0% and 9.8% of pregnancies with oligohydramnios are associated with chromosomal anomalies and renal anomalies, respectively (29). The gestational age influences oligohydramnios management. If discovered after 37 wk of gestational age, it cannot induce premature labor. But it causes a significant increase in the risk of the fetus needing an operative delivery. It is increased the induction rate (56% ) and cesarean rate (57%), as well as a higher risk of perinatal mortality (30).

An AFV of greater than 2000 ml, detected by ultrasound, indicates polyhydramnios (31). Chromosomal abnormalities, non-immune hydrops, twin-twin transfusion syndrome, and diabetes can cause polyhydramnios (32). Pregnancies with polyhydramnios are associated with a higher risk of premature birth, macrosomia, non-reactive non-stress tests, cesarean delivery, perinatal morbidity, and congenital anomalies (33). The rate of congenital malformations in polyhydramnios is 2.3% compared to 0.13% in cases of normal AF. Congenital malformation increases the risk of respiratory distress and hypoglycemia (34).

Polyhydramnios is related to fetal macrosomia and maternal pre-gestational/gestational diabetes (35). Polyhydramnios can be managed by sonographic evaluation for anatomical causes, TORCH serology testing, evaluation for maternal diabetes, and consideration of isoimmunisation if it is detected early. All the above should be considered, excluding an anatomical evaluation, if polyhydramnios is detected late (36).

### Amniotic fluid stem cells (AFSCs)

AF is bordered by the amniotic membrane and the embryo's skin on one side, and the embryonic urinary tract, digestive tract, and respiratory ducts on the other side. AF has different embryonic cells from the 3 embryonic germinal layers (37). The fetal origin of these cells is represented by karyotype analysis (38). AF has fully differentiated cells, precursor and multipotent stem-like cells (39).

In 1993, hematopoietic progenitor cells were found in the AF before the 12 ${}^{\text{th}}$ wk of gestation. In 1996, stem cells were found to be present in AF when they were cultivated in the supernatant of rhabdomyosarcoma cell lines and expressed dystrophin as a skeletal muscle protein (40).In 2003, mesenchymal stem cell markers' (Cluster of Differentiation (CD 90), CD105, CD73, CD44) expression of AF-derived cells was confirmed. These can differentiate into osteocytes, adipocytes, and fibroblasts (41). Prusa et al. demonstrated that AF-derived cells express Oct-4 similar to embryonic carcinoma cells, embryonic stem cells, and embryonic germ cells (42). In 2015, it was confirmed that some AF cells activate Rex-1 promoters like undifferentiated embryonic stem cells (43). In 2009, CD117 (c-Kit) immunoselection marker expression in AFSCs was detected (44). This marker plays a significant role in melanogenesis, gametogenesis and hematopoiesis, and presents on primordial germ cells, human embryonic cells and many somatic stem cells (45).

### Characteristics of AFSCs

AFSCs grow easily, have extensive self-renewal capabilities and appear phenotypically and genetically stable in culture (46). They can present with various morphologies from a fibroblast-like shape to an oval-round shape. They possess great proliferative properties with an average of over 250 population doublings in 36 hr and the clonogenic potential (86 ± 4.3 colonies) in culture (47).

Early pregnancy (first trimester) human AFSCs have 82% phenotype homology in transcriptome identity with embryonic stem cells (48). They are a heterogeneous population of 8-15 μm size. Like pluripotent and embryonic stem cells, the smaller cells make colonies in culture and represent c-Myc, octamer-binding transcription factor 4 (Oct-4), Homeobox transcription factor (Nanog), Kruppel like factor 4 (Klf4), sex determining region Y (SRY), SRY-box 2 (Sox2), Stage-specific embryonic antigen-4 (SSEA4), T cell receptor alpha-60 (Tra-1-60), SSEA3, Tra-1-8, and alkaline phosphatase (ALP) expression. The primordial germ cell origin of first-trimester AFSCs has been confirmed with CD117, SSEA3, c-Kit, FGF-8, DAZL, NANOS, VASA, SSEA1, Sox17, STELLA, FRAGILIS, and PUM2 expression (49). As well as, first-trimester AFSCs present high levels of mesenchymal stem cell markers expression including CD29, CD73, CD44, CD90, and CD105. Human leucocyte antigen (HLA) expression of these cells is low for class I and negative for class II. They form embryoid bodies in vitro but do not form teratomas when injected in immunocompromised mice (50).

Second- and third-trimester human AFSCs show c-Myc, Oct-4 and SSEA4 expression while, they do not have Nanog, Klf4 SSEA3, Tra-1-60, Tra-1-81, or ALP expression. CD44, CD29, CD73, CD105, CD90, CD166, CD146 as MSC markers are expressed at high levels in AFSCs. These cells express HLA class I (HLA-ABC), but they do not express HLA class II (HLA-DR). When second-trimester AFSCs are transplanted in immunocompromised mice, they cannot form embryoid bodies and do not form teratomas. They show a low rate of chromosomal instability in both early- and long-term culture in vitro (51).

Similar to first-trimester AFSCs, 2^nd^-trimester AFSCs are of fetal origin and express migratory primordial germ cell markers (52). Any expression of hematopoietic markers CD45, CD34, CD31 and CD14 are not detected in AFSCs of any gestational age (43, 53). Second-trimester AFSCs can differentiate into bone, cartilage, muscle, fat, hepatic or neural stem cells, and dopaminergic neurons. The characteristics of AF cells are varied based on gestational age (54). Hematopoietic progenitor cells in AF can be initially obtained from a woman who is 12 wk pregnant. These cells probably originate from the yolk sac (55). During wk 14 of pregnancy, pluripotent stem cells are detected in the AF (42). From the 19 ${}^{\text{th}}$ wk, the AF contains a population of mesenchymal stem cells that has the ability to differentiate into adipocytes, neurons, and osteocytes with a high proliferation rate (56). Neural progenitor cells are detected at wk 15-17 of pregnancy (57). The number of AF cells increases with gestational age, while in pathological conditions such as urogenital atresia and spina bifida low counts and extra counts occur, respectively (Table III) (58-62).

**Table 3:** Gene expression profile of AFSCs


**Author, year (Ref)**	**Markers**	**Antigen or CD**	**First trimester**	**Second and third trimester**
	Oct4	+ +
	Nanog	+ ${}^{\_}$
	Sox2	+ ${}^{\_}$
	SSEA4	+ +
	c-Myc	+ +
	Tra-1-60	+ ${}^{\_}$
	SSEA3	+ ${}^{\_}$
	Tra-1-81	+ ${}^{\_}$
	ALPL	+ ${}^{\_}$
**Loukogeorgakis ** ***et al.*** **, 2017**	Stem cell	Klf4	+ ${}^{\_}$
**Cananzi ** ***et al.*** **, 2009**		CD29	+ +
**Guillot ** ***et al.*** **, 2007 **		CD44	+ +
**Baghaban Eslaminejad ** ***et al.*** **, 2012 (59-62)**		CD73	+ +
		CD90	+ +
		CD105	+ +
		CD166	+ +
		Mesenchymal	CD146	+ ${}^{+}$
			CD45	${}^{\_}$	${}^{\_}$
			CD34	${}^{\_}$	${}^{\_}$
	CD31		${}^{\_}$	${}^{\_}$
	Hematopoietic		CD14	${}^{\_}$	${}^{\_}$
			HLA class I	${}^{+}$	${}^{+}$
			MHC	HLA class II	${}^{\_}$	${}^{\_}$
AFSCs: Amniotic fluid stem cells, CD: Cluster of differentiation, Oct4: Octamer-binding transcription factor 4, Nanog: Homeobox transcription factor nanog, Sox2: Sex determining region Y-box 2, SSEA4: Stage-specific embryonic antigen-4, c-Myc: A family of regulator genes and proto-oncogenes that code for transcription factors, Tra-1-60: A cell surface antigen, expressed along with SSEA-3, SSEA-4 and TRA-1-81 in human embryonic stem cells, SSEA3: Stage-specific embryonic antigen 3, Tra-1-81: A cell surface antigen expressed along with SSEA-3, SSEA-4 and TRA-1-60, ALPL: Provides instructions for making an enzyme called tissue-nonspecific alkaline phosphatase, Klf4: Kruppel like factor 4, MHC: Major histocompatibility complex, HLA: Human leucocyte antigen				

### AFSCs in tissue engineering and regeneration

Tissue engineering is an interdisciplinary field of clinical applications and research focused on injured cells, tissues, and organ regeneration (63). In this discipline, scaffolds seeded with cells are used for the regeneration of damaged tissues (64). Recruiting cells for transplantation restrains proliferation and differentiation of somatic differentiated cells (65). Also, adult stem cells may maintain epigenetic modifications after reprogramming, which may limit their application (59). Stem cells isolated from embryonic and fetal tissues may facilitate these major advancements (65). These cells are safe for clinical application given their genetic stability, differentiation capacity, and the epigenetic control system of early-passage AFSCs (66). Thus, AF is an important source of stem cells both in basic research and for regenerative medicine purposes. Engineered constructs using AFSCs promote the regeneration of damaged tissues (Table IV) (67-77).

**Table 4:** Differentiation potential of human amniotic epithelial stem cells


**Author, year (Ref)**	**Regenerated tissue**	**Treatment**
**Kajiwara ** ***et al*** **., 2017, Kunisaki, 2018 (67, 69)**	Fetal	Congenital diaphragmatic hernia, abdominal wall defects, spinal bifida, and congenital heart
**Cipriani ** ***et al*** **., 2007 (68)**	Peripheral and central nervous	Neurodegenerative disease such as cerebral ischemia
**Di Baldassarre ** ***et al*** **., 2018 (70)**	Cardiac	Cardiomyoplasty
**Chun ** ***et al.*** **, 2014 (71)**	Skeletal muscle	Duchenne muscular dystrophy
**Carraro ** ***et al*** **., 2008 (72)**	Lung	Lung-related disorders such as COVID-19
**Morigi ** ***et al*** **., 2014 (73)**	Kidney	Acute ischemia-reperfusion and acute tubular necrosis
**Wang ** ***et al*** **., 2018 (74)**	Hepatic	Liver fibrosis
**Maraldi ** ***et al*** **., 2013 (75)**	Bone and cartilage	Extensive bone defects disease
**Chang ** ***et al*** **., 2018 (76)**	Ovarian	Ovarian dystrophy and fertility
**Gosemann ** ***et al*** **., 2012 (77)**	Inflammatory and autoimmune diseases	Graft-vs.-host disease, inflammatory bowel disease, experimental autoimmune encephalomyelitis, and systemic lupus erythematosus

### Fetal tissue reconstruction

Early in fetal life, aberrant organogenesis can be caused by structural birth defects such as congenital diaphragmatic hernia, abdominal wall defects, congenital diaphragmatic hernia spinal bifida, congenital heart disease and spinal bifida. Using progenitor cells derived from AF is a novel therapeutic approach to organ regeneration in these pediatric diseases (67, 69). Diaphragmatic defect in neonatal lambs can be repaired with the mesenchymal amniocyte-based construct. Seeding a subpopulation of AFSCs onto polyglycolic acid/poly-4-hydroxyapatite, type I collagen, or polypropylene scaffolds could be a good construct for tissue-engineered diaphragmatic regeneration (78). AFSCs are preferred sources for fetal tissue reconstruction.

### Neural tissue regeneration

AFSCs have been reported to be able to promote regeneration in central and peripheral nervous tissue. AFSCs become positive for nestin in a first induction phase and through time a fraction of the cells express dopaminergic markers and present a pyramidal morphology (79). AFSCs can be differentiated into the neuro-ectodermal lineage, exhibiting a barium-sensitive potassium current, expressing neuronal markers (e.g. GIRK potassium channels), and releasing glutamate after stimulation (80).

AFSCs can differentiate and migrate to a variety of brain regions including the hippocampus, periventricular areas, and the olfactory bulb when these cells are implanted into the lateral cerebral ventricles of neurodegenerative model mice brains. When this has been studied, they are indistinguishable from surrounding murine cells (65). AFSCs that have been transplanted into the striatum of normal and ischemic rats have differentiated into neurons as well as astrocytes (68). AFSCs in fibrin glue or matrigel have been delivered into the crushed sciatic nerve where they promoted peripheral nerve regeneration (81). AFSCs promisingly promote peripheral and central nerve regeneration.

### Cardiac regeneration

AFSCs can be differentiated into smooth muscle (smoothelin) cells, endothelial (angiopoietin, CD146), and cardiomyocyte (Nkx2.5, MLC-2v, GATA-4, β-MyHC) when implicated in cardiomyoplasty (70). AFSCs grow and proliferate, induce higher vascular density, promote attenuation of left ventricular remodeling, and thus lead to an improvement in cardiac function when implanted in the infarcted area of model mice (82). Transplantation of a cell sheet fragment composed of AFSCs and thermoresponsive methylcellulose extracellular matrices into the peri-ischemic area of an immune-suppressive rat model, remarkably enhanced vascular density, recovered wall thickness, and also substantially decreased the infarct size (83). Transplanted AFSCs can improve cardiac function.

### Skeletal muscle regeneration

Differentiation of AFSCs into skeletal myogenic cells and skeletal muscle regeneration has been confirmed. These cells express desmin, troponin I, and α-actinin as skeletal cell-specific markers in vitro(84-86). Transplantation of the cells into cardiotoxin-injured mice has demonstrated that they differentiate into myogenic precursor cells and aggregate with host myofibrils (87). Transplantation of the muscle progenitor cells that differentiated from AFSCs into the denervated external urethral sphincter enhances urodynamic action and expression of myogenic-related markers (71). These findings demonstrate that AFSCs are one of the most hopeful cells to treat skeletal muscle degenerative diseases such as Duchenne muscular dystrophy.

### Lung epithelial regeneration

Transplantation of human AFSCs into an injured murine lung has been shown to cause differentiation into pulmonary lineages that express bronchiolar and alveolar markers and are associated with lung mucosal cells (72). It seems that AF is a valid resource for cell-based therapy for lung-related disorders.

### Kidney regeneration

AFSCs can differentiate to renal fate in an ex vivo system. When they are transplanted into a mouse embryonic kidney they aggregate with the renal tissue, take part in nephrogenesis, and express molecular markers apecific for primary kidney differentiation like ZO-1, GDNF, and claudin (88). Injection of the cells into a damaged kidney has been shown to lead to ameliorates acute tubular necrosis in a mouse cognate model (73). When nephrectomy model rats were treated with AFSCs, a remarkable decrease was seen in proteinuria, macrophages, blood pressure, and expression of α-smooth muscle actin (89). AFSCs with renal progenitor phenotype represent nephroprotective effects in model of acute ischemia-reperfusion. Treatment by AFSCs can cause a substantial decrease in serum creatinine level, hyaline cast formation, myofibroblasts, inflammatory cell infiltration, and tubular necrosis, and can increase the proliferation index (79).

### Hepatic regeneration

AFSCs can be induced to differentiate into hepatocyte cells that express albumin, hepatocyte nuclear factor 4, alpha-fetoprotein, hepatocyte growth factor receptor, and the multidrug resistance membrane transporter MDR120, and secrete urea (90). Transplantation of AFSCs into liver fibrosis mouse models has revealed that the cells fuse with the recipient liver cells and improve hepatic function (74).

AFSC transplantation into fulminant hepatic failure mouse models can improve liver function and increase survival rates (91). AF is a promising origin of progenitor cells for liver cell transplantation and hepatocyte regeneration.

### Bone and cartilage regeneration

AFSCs have been used to develop bone constructs using tissue engineering principles. The cells are cultured within the scaffold differentiated into osteoblastic or chondrocyte cells (92, 93). Differentiated cells produce mineralized calcium, and express and secrete alkaline phosphatase (ALP) as a surface marker of osteoblasts (94). AFSCs cultured within scaffolds such as grade poly-epsilon-caprolactone, poly-ε-caprolactone, poly-lactide-co-glycolide, fibrin, nonoscaffold, alginate/collagen, and combination of collagen, poly-D, L-lactic acid, and silk fibroin produce 3D mineralized high-density tissue-engineered bone both in vitroand in vivo (75). AF has been validated as an effective source for functional repair of extensive bone defects.

### Ovarian regeneration

AFSCs have displayed important roles in the area of reproductive health. Derivation of oocyte-like cells from AFSCs with high efficiency present a new approach to the check of human germ cell development in vitro (95). These cells express germ cells' markers and restore folliculogenesis in germ cell-ablated mouse models (96). They can alleviate bovine ovarian dystrophy and restore fertility (76).

### Regeneration of inflammatory and autoimmune diseases

Immunomodulatory effects of stem cells have been confirmed in different disease models such as graft-vs.-host disease, inflammatory bowel disease, experimental autoimmune encephalomyelitis, and systemic lupus erythematosus. AFSCs have remarkable immunomodulatory properties; they can be used for immunomodulatory cell therapy in autoimmune diseases and inflammatory conditions (77). They can prevent proliferation of T and B cell, macrophages, repress inflammatory properties of monocytes, neutrophils, natural killer cells and dendritic cells, and regulate the rule of T cells and anti-inflammatory M2 macrophages. These properties have enabled their use for the treatment of inflammatory and autoimmune-based diseases (97).

### COVID-19

COVID-19 can cause an aggressive inflammatory and fibrotic response in both lungs. AF in the lungs of the unborn baby developed the lung-specific mesenchymal stem cells during pregnancy. This cells can be important for treating lung disease. A phase 1 trial for assessing the efficacy AFSCs to treat COVID-19 has been started by Monash Health researchers (98).

### Clinical trial phase of AFSCs

Plasticity and immunomodulatory properties, of AFSCs have been candidates for treatment of different diseases. Previous preclinical studies have represented the AFSCs therapeutic potential in regenerative medicine. As of March 2021, 17 clinical trials about using these cells for curing different kinds of diseases were reported in the clinical trial databases Clinical Trials.gov and anzctr.org.au (Table V) (98).

**Table 5:** Clinical trials utilizing AFSCs


**Registration code**	**Disease**	**Results**	**Starting date**
**NCT00344708**	Damaged ocular surfaces	Re-establish the integrity of ocular surface	2006
**NCT02961712**	Myelopathy	Improvement of Osame's Motor Disability score	2016
**NCT03107975**	Spastic cerebral palsy	Improvement in gross motor function	2017
**NCT04414813**	Parkinson's disease	Dopaminergic neurons differentiation	2019
ACTRN12618000076279*	Ischaemic stroke	Reducing infarct volume	2019
**NCT02912104**	Primary ovarian insufficiency	Transdifferentiated into granulosa cells	2016
**NCT03223454**	Asherman's syndrome	Improvement in endometrial thickness and fertility	2017
**NCT03207412**	Ovarian insufficiency	Improvement in mice fertility	2017
**NCT03381807**	Refractory severe intrauterine adhesion	Improvement in endometrial thickness and fertility	2017
**NCT02959333**	Bronchial fistula	Recovery of bronchial fistula	2016
**ACTRN12614000174684**	Bronchopulmonary dysplasia	Rescue lung injury	2014
ACTRN12620000676910 p**	COVID-19-related respiratory failure	Improved lung dysfunction	2019
**NCT03031509**	Nonunion of limb fracture	Osteogenic differentiation	2017
**NCT03764228**	Acute graft-vs.-host disease	Preventing acute graft-vs.-host disease	2018
**ACTRN12616000437460**	Hepatic fibrosis	Reduce liver fibrosis and stimulate regeneration of liver cells	2017
**ACTRN12618001883202**	Crohn's disease	Change in nature, severity or frequency of disease	2018
AFSCs: Amniotic fluid stem cells,*the cell source was human amniotic epithelial stem cells except the specified reference. Which was human amnion stem cells. **All clinical trials was in 1 ${}^{\text{st}}$ stage except the specified reference which was the 2 ${}^{\text{nd}}$ stage			

## 2. Discussion

After the 10 ${}^{\text{th}}$ -20 ${}^{\text{th}}$ gestation wk, free diffusion happens between the fetus and AF through the placenta, fetal skin, and umbilical cord (4). At first, AF is mainly composed of water from the mother's body (98%) and the remaining 2% is salt and cells from the baby. But after the 12 ${}^{\text{th}}$ -14 ${}^{\text{th}}$ wk, it is also composed of carbohydrates, proteins, lipids, phospholipids, growth factors, and urea, which after the 12 ${}^{\text{th}}$ -14 ${}^{\text{th}}$ wk that help the development of the fetus (99). During this period, AF composition is similar to that of fetal plasma, thus the analysis of AF composition can provide useful would represent worth information about of the pathological or physiological status of the fetus (1, 100).

AF also contains comprises a heterologous cell population from all 3 embryonic germ layers (101). It has been proposed stem cells that the biological and therapeutic characteristics of AF stem cells were be studied (99). These cells can be easily obtained during amniocentesis with low risk for the mother and the fetus, and grow in vitro(89).

AFSCs have genetic stability, non-tumorigenicity, anti-inflammatory properties, high proliferative capacity, angiogenic potential, growth factor secretion, immunomodulatory properties and low immunogenic activity. Studying AFSCs does not pose any ethical concerns for recruiting participants in in vivo experiments.

Therefore, they serve as a promising source of cells for tissue engineering applications in regenerative medicine (102). These cells can regenerate several tissues faults, such as fetal tissue, heart, the nervous system, kidneys, lungs, cartilage, bones, and ovary tissues. Accordingly, AFSCs are becoming a promising tool for the treatment of a wide range of congenital and acquired human disorders (99, 103).

## 3. Conclusion

In this review, we described AF as a fluid that surrounds the baby in the uterus. We explained the sources of AF and its vital functions in fetal development. We illustrated that it is composed of nutrients, hormones, and biochemical products. The appearance and volume of AF depend on gestational age. Finally, the survival of stem cells in the AF, their high proliferation rate, the substantial potential of differentiation, normal karyotype, and low immunogenicity were discussed. We elaborated on the potential of AFSCs to promote the regeneration of various tissue defects in vitro and in vivo.

##  Conflict of Interest 

The authors declare that there is no conflict of interest.
